# UV-Mediated Photofunctionalization of Indirect Restorative Materials Enhances Bonding to a Resin-Based Luting Agent

**DOI:** 10.1155/2021/9987860

**Published:** 2021-05-29

**Authors:** Kyoko Ishikawa, Monica Yamauti, Antonin Tichy, Masaomi Ikeda, Takeshi Ueno, Noriyuki Wakabayashi, Ornnicha Thanatvarakorn, Taweesak Prasansuttiporn, Celso Afonso Klein-Junior, Akifumi Takahashi, Tomohiro Takagaki, Masatoshi Nakajima, Junji Tagami, Keiichi Hosaka

**Affiliations:** ^1^Department of Cariology and Operative Dentistry, Graduate School of Medical and Dental Sciences, Tokyo Medical and Dental University, 1-5-45, Yushima, Bunkyo-ku, Tokyo 113-8549, Japan; ^2^Department of Restorative Dentistry, Graduate School of Dental Medicine, Hokkaido University, Kita 13, Nishi 7, Sapporo 001-0012, Japan; ^3^Institute of Dental Medicine, First Faculty of Medicine of the Charles University and General University Hospital in Prague, Karlovo namesti 32, Prague 121 11, Czech Republic; ^4^Oral Prosthetic Engineering, Graduate School of Medical and Dental Sciences and Technology, Tokyo Medical and Dental University, 1-5-45, Yushima, Bunkyo-ku, Tokyo 113-8549, Japan; ^5^Department of Removable Partial Prosthodontics, Graduate School of Medical and Dental Sciences, Tokyo Medical and Dental University, 1-5-45, Yushima, Bunkyo-ku, Tokyo 113-8549, Japan; ^6^Faculty of Dentistry, Bangkok Thonburi University, 16/10 Taweewatana, Bangkok 10170, Thailand; ^7^Department of Restorative Dentistry and Periodontology, Faculty of Dentistry, Chiang Mai University, Center of Excellence in Materials Science and Technology, Chiang Mai University, T. Suthep, A. Muang, Chiang Mai 50200, Thailand; ^8^Department of Operative Dentistry, Lutheran University of Brazil, 301, Cachoeira do Sul, Canoas, RS 96501-595, Brazil; ^9^General Dentistry 1, The Nippon Dental University Hospital, 2-3-16 Fujimi, Chiyoda-ku, Tokyo 102-8158, Japan; ^10^Department of Operative Dentistry, Division of Oral Functional Science and Rehabilitation, School of Dentistry, Asahi University, 1851 Hozumi, Mizuho, Gifu 501-0296, Japan; ^11^Department of Regenerative Dental Medicine, Tokushima University Graduate School of Biomedical Sciences, 3-18-15 Kuramotocho, Tokushima 770-8504, Japan

## Abstract

**Purpose:**

The potential of UV-mediated photofunctionalization to enhance the resin-based luting agent bonding performance to aged materials was investigated.

**Methods:**

Sixty samples of each material were prepared. Yttria-stabilized zirconia (YZr) and Pd-Au alloy (Pd-Au) plates were fabricated and sandblasted. Lithium disilicate glass-ceramic (LDS) was CAD-CAM prepared and ground with #800 SiC paper. Half of the specimens were immersed in machine oil for 24 h to simulate the carbon adsorption. Then, all of the specimens (noncarbon- and carbon-adsorbed) were submitted to UV-mediated photofunctionalization with a 15 W UV-LED (265 nm, 300 mA, 7692 *μ*W/cm^2^) for 0 (control groups), 5, and 15 min and subjected to contact angle (*Ɵ*) measurement and bonded using a resin cement (Panavia™ V5, Kuraray Noritake, Japan). The tensile bond strength (TBS) test was performed after 24 h. The *Ɵ* (°) and TBS (MPa) data were statistically analyzed using two-way ANOVA and Bonferroni correction tests (*α* = 0.05).

**Results:**

In the carbon-adsorbed groups, UV-mediated photofunctionalization for 5 min significantly decreased *Ɵ* of all materials and increased TBS of YZr, and UV for 15 min significantly increased the TBS of LDS and Pd-Au. In noncarbon-adsorbed groups, UV-photofunctionalization did not significantly change the *Ɵ* or TBS except YZr specimens UV-photofunctionalized for 15 min.

**Conclusion:**

UV-mediated photofunctionalization might have removed the adsorbed hydrocarbon molecules from the materials' surfaces and enhanced bond strengths of Panavia™ V5 to YZr, LDS, and Pd-Au. Additionally, UV-mediated photofunctionalization improved the overall TBS of YZr. Further investigation on the optimum conditions of UV photofunctionalization on indirect restorative materials should be conducted.

## 1. Introduction

In recent years, indirect tooth-colored restorative materials such as zirconia and glass-ceramics have been widely used due to their esthetics and increased patients' demand for natural color restorations. Still, in some clinical cases, restorations fabricated with precious metal alloys are necessary. Moreover, based on the concept of minimal intervention dentistry, the development of restorations with higher mechanical strength, better esthetics, and improved bonding performance of resin-based luting agents has enabled minimum tooth preparation placement [1].

The effect of aging, that is, the effect of time after processing the titanium surface, is crucial to the implants' osteoconductivity. Titanium surfaces adsorb hydrocarbons progressively overtime under ambient conditions, which in part determines titanium's surface energy [2]. The atmosphere also modifies other metals and ceramics' exposed surfaces by producing this carbon-containing layer [3–6]. Hydrocarbon molecules' production is unavoidable due to the constant accumulation of carbonyl moiety, particular hydrocarbons from the atmosphere and surrounding environment during the implants' preparation and storage, and indirect restorations placement [7].

When the indirect dental restorative materials are kept and stored in the atmosphere, water and organic molecules which contain carbon are adsorbed on their surface [3–6]. Hydrocarbons can also be adsorbed on zirconia surfaces, leading to increasing contact angle due to the adsorbed hydrocarbons' lower surface energy [8]. For nonpolar chemical species without nonbonding electron pairs such as alkanes (C_n_H_2n+2_), physical carbon adsorption on the zirconia surface has been observed [9]. Furthermore, CO_2_ is most commonly adsorbed onto zirconium oxide (or zirconia) as the carbonate form [10]. Therefore, as it occurs in implant surfaces, there might be a risk that the cleaned restorative surfaces may be aged from the laboratory's bench to the dental office, which may compromise the bonding performance of the surfaces to a resin-based luting agent, leading to future degradation of restoration-luting agent dentin. This speculation and the lack of data to support it led to the present investigation.

The ultraviolet (UV) photofunctionalization was initially used in titanium surfaces to control the biological and time-related aging from the processing or manufacture [2, 11, 12] to the implants' use. The UV photofunctionalization aims to decompose and remove the hydrocarbon molecules and restore the titanium's surface hydrophilicity under appropriate conditions, enhancing bone-titanium integration [7, 12–14]. More recently, the UV-mediated photofunctionalization has also been applied to orthodontic miniscrews [15, 16], zirconia oxide implants [17–20], gold [6], and other metal alloys as CoCrPd alloy [5] to promote hydrocarbons removal, increase the surface free energy, and stimulate the biointegration and osteoconductivity [17].

However, to the best of our knowledge, few studies have investigated the photofunctioning of aged indirect restorative materials mediated by UV irradiation from the viewpoint of adhesive bonding performance. Therefore, the study evaluated the bond strength of a resin-based luting agent to zirconia, lithium disilicate ceramics, and a Pd-Au alloy after UV-mediated photofunctionalization. The feasibility of using a new method to adsorb carbon to surfaces and a prototype of a UV-LED unit was also investigated. The null hypotheses were that UV photofunctionalization of the indirect restorative materials would not affect (1) the contact angle of the surfaces and (2) the tensile bond strength to a resin-based luting agent.

## 2. Materials and Methods

### 2.1. Study Design and Materials

This was a prospective laboratory study in which different materials were evaluated separately. The independent variables were surface contamination (noncarbon-adsorbed or carbon-adsorbed) and UV-light photofunctionalization time (0, 5, and 15 min). The outcomes were contact angle and tensile bond strength.

The materials, their basic composition, and manufacturers' instructions used in the study are described in Table 1. The used restorative materials were yttria-stabilized zirconia ceramic (YZr; TZ-3Y-E; Tosoh, Tokyo, Japan), lithium disilicate ceramic (LDS; IPS E-max CAD; Ivoclar Vivadent, Schaan, Liechtenstein), and a Pd-Au alloy (Pd-Au, Castmaster 12S; IDS, Tokyo, Japan).

### 2.2. Specimen Preparation

For each restorative material, sixty specimens, custom-made plates (10 × 10 × 2 mm), were prepared in a dental laboratory. After YZr sintering, LDS glazing, and Pd-Au casting, the specimens were used in two weeks. Before the specimen preparation, the adherent surfaces of YZr and Pd-Au surfaces were air-abraded with 50 *μ*m Al_2_O_3_ particles from a 10 mm distance for 20 s at 0.2 MPa and 0.4 MPa pressure respectively, using a blasting machine (Basic Master, Renfert, Hilzingen, Germany), and those of LDS were ground with 800-grit SiC paper (Sankyo Fuji Star, Saitama, Japan) under running tap water. Subsequently, all restorative materials were ultrasonically cleaned in distilled water for 5 min and dried with oil-free compressed air. Half of the specimens of each material were submitted to carbon adsorption simulation by storing them in a device containing oil composed of various types of hydrocarbons (CxHy) (Yoshida Spray, The Yoshida Dental MFG. Co., LTD., Tokyo, Japan) for an additional period of 24 h, dried with a dental three-way syringe, and were referred as *carbon-adsorbed groups*. All the specimens were submitted to UV-mediated photofunctionalization using a portable 15 W UV-LED light generator prototype (UV LED light, 265 nm, 300 mA, 7692 *μ*W/cm^2^, Nikkiso, Tokyo, Japan) at 1 cm for 0 (*control groups*), 5, and 15 min. Those groups that were not previously submitted to carbon adsorption simulation were named *noncarbon-adsorbed groups* after being UV-mediated photofunctionalized.

### 2.3. Contact Angle (Ɵ) Measurement

The surfaces of the specimens were prepared as described above. A standardized droplet of deionized water (1 *μ*L) was placed on the surface of each materials' specimen, and the contact angle (*Ɵ*) was measured using a video contact angle system (VCA OPTIMA XE, AST Products. Inc., MA, USA) at room temperature 25°C and humidity 50 ± 10%. The droplet's surface was monitored continuously, and the contact angle was measured just after 20 s when the droplet was stabilized [21]. Each measurement (°) was taken in sextuplicate for each specimen (*n* = 20).

### 2.4. Tensile Bond Strength (TSB) Test

After the contact angle measurements, the surfaces of the materials were covered with a polyethylene film (100 *μ*m thickness) with a circular hole (diameter 4 mm), in which the Ceramic Primer Plus (Kuraray Noritake Dental Corp., Japan) and the resin-based luting agent (Panavia™ V5, Kuraray Noritake Dental Corp., Japan) were applied as per the manufacturer's instructions. A stainless steel metal rod previously sandblasted and treated with a metal primer (Alloy Primer, Kuraray Noritake Dental Corp.) was carefully and vertically pressed against the mixed luting agent, and excess of the material was removed off using a micro brush. The resin-based luting agent was light cured for 10 s from four sides using an LED light-curing unit (Valo Grande, 1200 mW/cm^2^, Ultradent, UT, USA). After the 30 min setting time, all specimens were stored in distilled water at 37°C for 24 h. After storage, the specimens were positioned in a testing apparatus with the bonded interface oriented perpendicularly to the tensile load and subjected to the TBS test at a crosshead speed of 0.5 mm/min (Autograph AGS-J, Shimadzu, Kyoto, Japan). After testing, the debonded interfaces were observed using a stereomicroscope (Nikon SMZ1000, Nikon, Tokyo, Japan) at a 35x magnification for failure mode classification. Failure modes were classified into three following categories: (1) adhesive failure at the interface between the materials and the resin cement, (2) cohesive failure within the resin cement, and (3) mixed failure combining adhesive and cohesive failures [21].

### 2.5. Statistical Analysis

The TBS and *Ɵ* data of each indirect restorative material were tested for normality and homoscedasticity. As the values followed a normal and homogeneous distribution, the data of both outcomes were statistically analyzed using a two-way ANOVA for each material (carbon − adsorption × duration of UV-mediated photofunctionalization), followed by a *t*-test with Bonferroni correction for pairwise comparisons. No comparisons between the materials were performed as this was not part of the research question. The analyses were performed at a significance level of 0.05 using the IBM SPSS Statistics 27 software (IBM Corp., Armonk, NY, USA).

## 3. Results

### 3.1. Contact Angle

The mean *Ɵ* and standard deviations of YZr, LDS, and Pd-Ag are presented in Table 2. Two-way ANOVA revealed that *Ɵ* values were significantly affected by carbon adsorption and UV photofunctionalization time as per each restorative material. There was a significant interaction between carbon adsorption and each material's UV application time. For all materials, in the carbon-adsorbed groups, the *Ɵ* significantly decreased as the UV application time increased. On the contrary, there were no significant differences among UV application time in the noncarbon-adsorbed groups of all restorative materials. For all materials, in the control groups (no UV application), the carbon-adsorbed groups presented higher *Ɵ* values than those of noncarbon-adsorbed groups.

For each material, different superscript letters indicate significant differences between groups: uppercase letters in a column and lowercase letters in the row; ^∗^ × ^∗∗^interaction between variables.

### 3.2. TBS

The mean TBS and standard deviations of Panavia™ V5 to UV-photofunctionalized YZr, LDS, and Pd-Au specimens are presented in Table 3. Two-way ANOVA revealed that UV-mediated photofunctionalization and application time significantly affected TBS for all materials. Moreover, there was a significant interaction between the independent variables in the case of LDS and Pd-Au. For noncarbon-adsorbed groups, UV-mediated functionalization for 15 min (35.2 ± 7.7 MPa) had a positive effect on the bond strength of YZr to Panavia™ V5 but did not improve the bonding of the other materials. On the other hand, the UV photofunctionalization significantly increased the TBS of carbon-adsorbed groups of all materials to the luting agent, mainly when 15 min application was performed. In the control groups (i.e., no UV-mediated photofunctionalization), the carbon adsorption harmed the bond strength of all materials to the resin-based luting agent, indicating the effect of the oil-storage method.

For each material, different superscript letters indicate significant differences between groups (*p* < 0.05): uppercase letters in a column and lowercase letters in a row; ^∗^ × ^∗∗^interaction between variables.

### 3.3. Failure Mode Analysis

**Figure 1:**
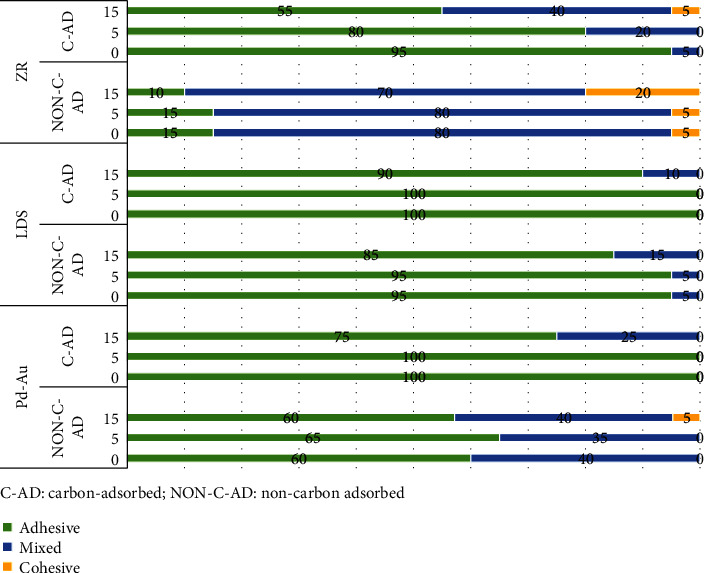
Failure mode (%) as per restorative materials and UV-photofunctionalization treatment and time.

Failure mode distributions of debonded specimens are shown in Figure 1. In the YZr specimens, the predominant failure mode was adhesive (interfacial) in carbon-adsorption groups, while mixed failure prevailed in the noncarbon-adsorbed groups. The extended UV-mediated photofunctionalization treatment decreased interfacial failures and increased both cohesive and mixed failures in both noncarbon-adsorbed and carbon-adsorbed groups. For LDS and Pd-Au, the predominant failure mode was adhesive (interfacial) failure in noncarbon-adsorbed and carbon-adsorbed groups. In carbon-adsorbed groups, neither cohesive nor mixed failures were observed except in 15 min UV mediate photofunctionalization noncarbon-adsorbed groups.

## 4. Discussion

The adsorption of carbon has been a recurrent issue in implantology and orthodontics as the surfaces of titanium implants, and orthodontic miniscrews suffer contamination with hydrocarbons, compromising their bioinductivity, cellular migration, and osteointegration [7, 22]. Moreover, the fixation of indirect materials to tooth substrates requires luting agents, being the resin-based ones widely used [23]. The resin-based luting agents require restorations and tooth substrates' physicochemical preparation and cleaning that are restorative- and luting agent-dependent [24]. The preparation of restorations' inner surfaces is a critical step in which high pressurized particle abrasion or strong acids (i.e., hydrofluoric acid—HF) can be used [23, 24]. However, in Japan, hydrofluoric acid cannot be used by dental clinicians since a 3-year-old child patient died of acute drug intoxication from hydrofluoric acid in 1982 when fluoride solution was misplaced by the acid [25]. Thus, nowadays, dentists cannot buy HF acid, and they rely on the dental technician for the HF application. Alternatively, the dentist would use phosphoric acid to etch before the silane coating is applied [25–27]. More recently, a hypersaturated solution of zirconium oxide particles in sodium hydroxide and water has been introduced and showed an effective decontamination potential to clean zirconium surface by removing its organic contents [28–30]). Still, there is a continuous search for alternatives to replace HF clinical practice, especially now that there is an increase in indirect restorations that demands surface pretreatment before luting.

This in vitro study showed that UV-mediated photofunctionalization reduced the contact angle of ceramics and metal, allowing a more appropriate spread of the primers [31, 32] and improved their bond strength. From a clinical perspective, the restorations were left two weeks to age (that is, to adsorb carbon naturally), and then the adherent surface was prepared to simulate the clinical situation. Therefore, both null hypotheses were rejected. The UV photofunctionalization might help replace the HF as a decontaminant agent, as demonstrated in implant and miniscrew surfaces [16, 22, 33]. Furthermore, due to the indirect restorations' occlusal adjustment and saliva contamination during try-in [34, 35], it is expected that dentists would return the restorations to the dental laboratory for repolishing of outer surfaces [36] and sandblasting of inner surfaces [34–36]. Consequently, an additional bench-chairside time lag could occur, jeopardizing the adherent's surface cleanliness.

YZr benefited the most from the treatment, as even 5-minute UV photofunctionalization significantly increased its bond strength. This finding agrees with the previous study reporting that UV treatment to tetragonal zirconia polycrystal (as the one used in this study) remarkably decreased the carbon content and increased the surface's hydrophilicity [37, 38]. Regardless of adherent material, 15 min of UV-mediated photofunctionalization of the carbon-adsorbed specimens promoted a significant decrease in the *Ɵ* (Table 2) and increased bond strength compared to the control groups (0 min) (Table 3). The contact angle decrease promoted more intimate interaction between the resin-based luting agent and the adherent, favoring the bond strength [37]. The increase of bond strength resulted in mixed and cohesive failures of carbon-adsorbed groups (Figure 1). The relation between decreased contact angle followed by increased adherent surface wettability and increased bond strength was extensively investigated [30, 39, 40].

In noncarbon-adsorbed groups, the influence of UV photofunctionalization on *Ɵ* and TBS was not observed in LDS and Pd-Au specimens. However, 15-minute UV treatment significantly increased YZr's TBS with increased cohesive failures; although, *Ɵ* values were not significantly changed. This finding may indicate that UV photofunctionalization could have an additional effect to enhance the YZr's bond strength compared to sandblasting followed by a 10-MDP containing primer only. Due to this effect of UV irradiation [18, 41, 42], stainless steel [43] and fiber posts [44] have improved these materials' bond strength to dentin.

Considering the control groups (non-UV-mediated), regardless of materials, the *Ɵ* of noncarbon-adsorbed specimens was lower, and TBS was higher than those results of carbon-adsorbed counterparts. These findings strongly suggested that the oil storage method could artificially adsorb carbon on the adherents' surfaces. Furthermore, the statistical differences between the control and UV photofunctionalized groups indicate that UV-irradiation positively affected those surfaces by removing impurities such as hydrocarbons and increasing their wettability [41, 42, 45].

The present study's strengths also rely on the proposed oil storage model to accelerate biological aging and the UV-mediated photofunctionalization device. A prototype of portable UV-LED equipment was employed in the study, aiming at the chairside or dental laboratory use. However, further research should address the chemical aspects of carbon adsorption in different indirect materials, using the oil storage model and the effect of the portable UV photofunctionalization. Studies on the optimum conditions (distance, duration of irradiation, power, absence of ozone) of UV-mediated photofunctionalization are also needed.

## 5. Conclusions

Within this study's limitation, it is suggested that UV-mediated photofunctionalization could decrease the contact angle of YZr, LDS, and Pd-Au surfaces and enhance the bond strengths to a resin-based luting cement.

## Figures and Tables

**Table 1:** Materials' type, name (abbreviation), manufacturer, elemental composition, and instructions of use.

Material type, name, (abbreviation), manufacturer	Basic composition	Instructions of use
Yttria-stabilized zirconia ceramic, TZ-3Y-E, (YZr), Tosoh, Tokyo, Japan	ZrO_2_, Y_2_O_3_, HfO_2_, Al_2_O_3_	—

Lithium disilicate ceramics, IPS e.max CAD (LDS), Ivoclar Vivadent, Schaan, Liechtenstein	SiO_2_, Li_2_O, K_2_O, P_2_O_5_, ZrO_2_, ZnO, Al_2_O_3_, MgO, coloring oxides	—

Precious metal alloy, Castmaster 12S, (Pd-Au), IDS, Tokyo, Japan	Au 12%, Pd 20%, Ag 54%, Cu 12%, additives	—

Alloy Primer, Kuraray Noritake, Japan	>90% acetone, 10-MDP	Roughen, wash with water, and dry the metal surface Apply alloy primer to the metal with a sponge and leave it for drying Prepare the tooth surface, core, or abutment Cement with Panavia™ V5 resin cement

Dual-cure resin luting agent, Panavia™ V5, (PV5), Kuraray Noritake, Japan	Paste A: Bis-GMA, TEGDMA, hydrophobic aromatic dimethacrylate, hydrophilic aliphatic dimethacrylate, initiators, accelerators, silanated barium glass filler, silanated aluminum oxide filler, colloidal silica Paste B: Bis-GMA, hydrophobic aromatic dimethacrylate, hydrophilic aliphatic dimethacrylate, initiators, accelerators, silanated barium glass filler, silanated aluminum oxide filler, accelerators, dl-camphorquinone, pigments	Attach a mixing tip or an endo tip to the syringe of Panavia V5 paste in the usual manner. Mix the pastes and apply the mixture over the entire adherent surface and place the metal device. Remove any excess cement *Nontranslucent* materials: allow the cement to chemical cure by letting it set for 3 min after placement of the prosthetic restoration. *Translucent materials:* light cure the entire surface of the prosthetic restoration using the dental curing unit. If the area to light cure is larger than the light-emitting tip, divide the exposure process into a few applications.

Clearfil™ Ceramic Primer Plus, Kuraray Noritake, Japan	10-MDP, *γ*-MTPS, ethanol	(1) Prepare the adherent (metal, zirconia, glass-ceramic, resin) (2) Dispense the necessary amount of Clearfil Ceramic Primer Plus into a well of the mixing dish immediately before application (3) Dry the entire adherent surface sufficiently using mild, oil-free airflow

ZrO_2_: zirconium dioxide; Y_2_O_3_: yttrium oxide; HfO_2_: hafnium oxide; Al_2_O_3_: aluminum oxide; SiO_2_: silicon dioxide; Li_2_O: lithium oxide; K_2_O: potassium oxide; P_2_O_5_: phosphorus pentoxide; ZnO: zinc oxide; MgO: magnesium oxide; Au: gold; Pd: palladium; Ag: silver; Cu: copper; VBATDT: 6-(4)vinyl benzyl-n-propyl)amino-1,3,5-trizaine-2,4-dithiol; 10-MDP: 10-methacryloyloxydecyl dihydrogen phosphate; Bis-GMA: bisphenol A-glycidyl methacrylate; TEGDMA: triethylene glycol dimethacrylate; *γ*-MTPS: *γ*-methacryloxypropyl trimethoxysilane.

**Table 2:** Mean(SD) (*°*) of the contact angle (*Ɵ*) according to the carbon adsorption condition^∗^ and UV-mediated photofunctionalization time^∗∗^.

Material	Surface condition	UV-mediated photofunctionalization time			Significance (*f*; *p*)
		0 min	5 min	15 min	
YZr	Noncarbon-adsorbed	34.2 (2.4)^aA^	32.8 (2.8)^aA^	34.1 (2.4)^aA^	^∗^7400.55; <0.001 ^∗∗^256.27; <0.001 ^∗^ × ^∗∗^ 261.95; <0.001
	Carbon-adsorbed	97.9 (2.7)^aB^	86.8 (3.9)^bB^	66.1 (3.9)^cB^	
LDS	Noncarbon-adsorbed	5.1 (1.3)^aA^	5.3 (1.3)^aA^	5.0 (1.5)^aA^	^∗^2732.23; <0.001 ^∗∗^21.70; <0.001 ^∗^ × ^∗∗^ 19.70; <0.001
	Carbon-adsorbed	32.3 (3.6)^aB^	29.1 (2.7)^bB^	25.1 (3.1)^cB^	
Pd-Au	Noncarbon-adsorbed	57.3 (2.3)^aA^	57.2 (2.4)^aA^	54.9 (4.1)^aA^	^∗^1190.10; <0.001 ^∗∗^108.90; <0.001 ^∗^ × ^∗∗^ 66.66; <0.001
	Carbon-adsorbed	87.5 (4.2)^aB^	80.6 (4.4)^bB^	67.3 (2.3)^cB^	

**Table 3:** Tensile bond strengths to various substrates: mean(SD) [MPa].

Material	Surface condition	UV-mediated photofunctionalization time			Significance (*f*; *p*)
		0 min	5 min	15 min	
YZr	Noncarbon-adsorbed	25.3 (4.5)^aA^	29.2 (5.7)^aA^	35.2 (7.7)^bA^	^∗^240.85; <0.001 ^∗∗^30.69; <0.001 ^∗^ × ^∗∗^0.26; 0.773
	Carbon-adsorbed	9.6 (4.1)^aB^	14.6 (4.7)^bB^	18.9 (4.5)^cB^	
LDS	Noncarbon-adsorbed	11.8 (3.6)^aA^	11.6 (3.6)^aA^	12.8 (4.1)^aA^	^∗^47.99; <0.001 ^∗∗^11.40; <0.001 ^∗^ × ^∗∗^5.56; 0.005
	Carbon-adsorbed	5.0 (2.5)^aB^	6.8 (3.0)^aB^	11.2 (3.4)^bA^	
Pd-Au	Noncarbon-adsorbed	16.7 (2.7)^aA^	17.0 (4.7)^aA^	18.5 (4.1)^aA^	^∗^210.08; <0.001 ^∗∗^22.74; <0.001 ^∗^ × ^∗∗^9.18; <0.001
	Carbon-adsorbed	5.1 (1.9)^aB^	6.7 (2.2)^aB^	13.1 (3.5)^bA^	

## Data Availability

The data used to support the findings of this study are included within the article.
